# Supplementary material for: Asynchronous magnetic resonance elastography: Shear wave speed reconstruction using noise correlation of incoherent waves
Source: Magn Reson Med. 2022 Oct 27;89(3):990–1001. doi: 10.1002/mrm.29502 (PMC9792433; doi:10.1002/mrm.29502)
Supplement: Supplementary file 1 — DATA S1 MATLAB code used to generate the numerical simulations of Figure 1 [file MRM-89-990-s002.zip › k-Wave/helpfiles/k-wave_getting_started.html]

Getting Started (k-Wave)


# Getting Started

## Run the First Example

Regardless of your intended application for the k-Wave Toolbox, the easiest way to get started is to work through the Initial Value Problems examples, in particular the Homogeneous Propagation Medium example. This gives a step-by-step introduction to the way the simulation functions within k-Wave work. Each of the examples comes with an accompanying m-file which can be opened or run from within the help menu. At the top of the help file for each example, links to earlier examples are also given ("It builds on..."). Components of the example that have been discussed in these earlier examples will generally not be explained again in detail.

After working through the Initial Value Problems, further examples are available specific to particular areas of interest, for example Time Varying Source Problems or Photoacoustic Image Reconstruction. Additional details can also be found within the help files for each function. These are listed both by category as well as in alphabetical order.

There is additional information on the functions and algorithms used in k-Wave in the k-Wave Manual (this can be downloaded from www.k-wave.org/documentation.php). The manual includes a general introduction to the governing equations and numerical methods used in the main simulation functions in k-Wave. It also provides a basic overview of the software architecture and a number of canonical examples. The manual has a different emphasis to the MATLAB documentation, thus it can be beneficial when starting with k-Wave to read both in parallel.

## Build Your Own Simulation

After running the various examples included within the k-Wave toolbox, it is straightforward to build your own simulation by using the existing examples as templates. To get started:

- open the list of examples,
- click on an example, eg. the Homogeneous Propagation Medium Example,
- click open the file to open the m-file in the MATLAB Editor, and
- save the file with a new name (in a directory that is on the MATLAB path) and make the changes you want.

For example, you could:

- change the size of the simulation grid,
- change the properties of the propagation medium,
- define a new initial pressure distribution,
- change the shape or type of sensor used to collect the data, or
- modify the default visualisation and computation options.

## Troubleshooting

If you are unable to run the examples or see the help files included within the k-Wave Toolbox, first check that the k-Wave Toolbox has been added to the MATLAB path. To add a directory to the MATLAB path, either use the `addpath` command, type `pathtool` on the command line, or select "Set Path" from the menus in the main MATLAB window. In each case, you should select the root k-Wave Toolbox folder to add to the path. Depending on your version of MATLAB, you may need to restart MATLAB before the help files will appear in the help browser.

If you are encountering problems using specific combinations of inputs using Octave or older versions of MATLAB, try turning the display off by setting the optional input parameter `'PlotSim'` to `false`, using a binary sensor mask or setting `'CartInterp'` to `'nearest'`, and using the default settings for `'DataCast'` (optional inputs are given as `'string', value` pairs following the `kgrid`, `medium`, `source`, and `sensor` inputs).

If you are still having problems go to the online k-Wave User Forum and search for an answer or post a question.
